# 4298 Prediction models for pulmonary tuberculosis treatment outcomes: a systematic review

**DOI:** 10.1017/cts.2020.138

**Published:** 2020-07-29

**Authors:** Lauren Saag Peetluk, Felipe Ridolfi, Valeria Rolla, Timothy Sterling

**Affiliations:** 1 Vanderbilt University Medical Center

## Abstract

OBJECTIVES/GOALS: Many clinical prediction models have been developed to guide tuberculosis (TB) treatment, but their results and methods have not been formally evaluated. We aimed to identify and synthesize existing models for predicting TB treatment outcomes, including bias and applicability assessment. METHODS/STUDY POPULATION: Our review will adhere to methods that developed specifically for systematic reviews of prediction model studies. We will search PubMed, Embase, Web of Science, and Google Scholar (first 200 citations) to identify studies that internally and/or externally validate a model for TB treatment outcomes (defined as one or multiple of cure, treatment completion, death, treatment failure, relapse, default, and lost to follow-up). Study screening, data extraction, and bias assessment will be conducted independently by two reviewers with a third party to resolve discrepancies. Study quality will be assessed using the Prediction model Risk Of Bias Assessment Tool (PROBAST). RESULTS/ANTICIPATED RESULTS: Our search strategy yielded 6,242 articles in PubMed, 10,585 in Embase, 10,511 in Web of Science, and 200 from Google Scholar, totaling 27,538 articles. After de-duplication, 14,029 articles remain. After screening titles, abstracts, and full-text, we will extract data from relevant studies, including publication details, study characteristics, methods, and results. Data will be summarized with narrative review and in detailed tables with descriptive statistics. We anticipate finding disparate outcome definitions, contrasting predictors across models, and high risk of bias in methods. Meta-analysis of performance measures for model validation studies will be performed if possible. DISCUSSION/SIGNIFICANCE OF IMPACT: TB outcome prediction models are important but existing ones have not been rigorously evaluated. This systematic review will synthesize TB outcome prediction models and serve as guidance to future studies that aim to use or develop TB outcome prediction models.

